# The start-up phase of a non-specialist paediatric surgical service by outreach focused on capacity building at the Consolata Hospital Ikonda, Makete District, Tanzania

**DOI:** 10.4102/phcfm.v12i1.2428

**Published:** 2020-10-22

**Authors:** Alessandro Calisti, Mariagrazia Andriani, Agnes Mlawa, Gian P. Zara

**Affiliations:** 1 Department of Pediatric Surgery, San Camillo Forlanini Hospital, Rome, Italy; 2 Ada Manes Foundation for Children, Pescara, Italy; 3 Department of Surgery, Consolata Hospital Ikonda, Makete, Tanzania; 4 Department of Medicine, Consolata Hospital Ikonda, Makete, Tanzania

## Abstract

**Background:**

The need for paediatric surgeons in Tanzania is largely unmet. Twelve in two main urban centres (0.06 for 100 000 population under 15 years of age) are far lower than the recommended workforce size. Complex conditions require a significant increase in the number of paediatric surgeons. In contrast, children with minor diseases, living in rural areas, could be managed even at the district level by trained general surgeons.

**Aim:**

The aim of this study was to develop capacity for general paediatric surgical services in a district hospital by outreach with a focus on mentorship.

**Methods:**

Capacity building priorities for non-specialist paediatric surgery were identified and addressed using evidence-based guidelines. Local general surgeons were involved in supervised clinical decision-making and in all surgical procedures. The visiting team provided daily meetings, weekly lectures, and on-job training. Electronic copies of recent surgical textbooks were provided together with video-conferencing distant specialist consultations.

**Results:**

A total of 715 children were handled by the visiting team during the 27-week period. Four hundred and fifty diseases were diagnosed amongst 406 children. Awareness of paediatric surgical needs, improved management of most common conditions like congenital hernias, undescended testis, hypospadias and anorectal malformations needing temporary colostomy occurred. Local general surgeons were assisted in treating 358 cases of general paediatric surgical conditions. Updated early management protocols were introduced for more complex diseases needing referral to specialist centres like solid tumours and neonatal abnormalities. The visiting team operated major paediatric surgical cases at the Consolata Hospital Ikonda.

**Lessons learnt:**

Surgical outreach and capacity building at the district hospital level could be a possible answer to the unmet paediatric surgical needs of children living in rural areas. Apprenticeship training for general surgeons may help to fill the gap provided that they are strongly motivated and supported on acquiring and implementing their paediatric surgical skills.

**Keywords:**

global health; paediatric surgery; humanitarian outreach.

## Introduction

More than 50% of the African population is in the paediatric age group, and 85% may need an operative procedure, generally minor, by the age of 15 years.^1^ If paediatric surgery is a well-recognised speciality in high-income countries (HICs) and specific standards and professional skills have been defined,^2^ the number of specialists in low-income countries (LICs) is still far to meet the needs.^3^

Although an academic paediatric surgical curriculum and training started in Tanzania since 2002 supported by international academic partnerships to fill the gap through various modalities (short courses, informal training for medical students, formal master-level training),^4,5^ the country has still a small paediatric surgical workforce. It was estimated to be, in 2019, only 12 surgeons working in two tertiary centres for a population of about 22 million under 15 years old.^3^

Most paediatric cases risk so far falling under the coverage of general surgeons not exposed to paediatric surgery as a specialist field during their undergraduate training and internship.^4^ They may be not always prepared to recognise that most of the paediatric diseases that they observe and treat are specific to this age group and pose quite separate problems of management. The quality of care may be severely affected.

Urgent solutions must be found in providing a better service, especially for children living in rural areas where poverty and lack of transports hamper access to specialist centres. Complex diseases must be distinguished from the more common conditions, and local general surgeons must be trained on safely referring the first to specialist hands and delivering to the second an appropriate treatment.^6^

The present study started with the interest of the Consolata Hospital Ikonda (CHI), a district health institution in Makete Region – Tanzania Board to expand and improve its children-oriented services by outreach from an Italian tertiary centre, focused on capacity building by an informal training on the job.

## Materials and methods

### Study setting

Consolata Hospital Ikonda is a 365-bedded private, not-for-profit hospital, which is located in the Southern Highlands of Tanzania. It provides both inpatient and outpatient paediatric services with a 33-bedded unit. Minor surgical procedures are performed on children and the hospital has no pediatric intensive care facilities.

The surgical workforce includes two general surgeons, one urology specialist together with eight clinical officers (COs) with a 3-year training and six assistant medical officers (AMOs). A partnership to support clinical activities was established in 2014 with the Catholic University of Rome, School of Medicine. Since many years volunteers from Europe (surgeons, orthopaedics, ENT, urologist, paediatricians) regularly visit the CHI assisting local staff with most complex surgical diseases. Paediatric surgeons joined them since 2016. Children’s surgery is sporadic, and local surgeons are occasionally exposed mainly to emergencies. Few elective procedures are usually performed.

### Study population

A total of 715 children were referred during outreach team visits from June 2016 to November 2019 for surgical consultations. Patients were classified into two categories: cases that did not require specialist skills and major cases needing a specialist approach.

### Intervention

Daily meetings for case revision and weekly lectures about diseases that providers found particularly difficult to manage, primarily congenital anomalies and tumours, helped to make ‘on the job’ surgical training more effective. Priorities for capacity building for non-specialist surgeons were identified and addressed according to evidence-based guidelines for most frequently encountered conditions and procedures.

Electronic copies of recent surgical textbooks were also provided. A distant specialist consultation based on video-conferencing technology was activated to assist local staff with complex scenarios when the visiting team was not at the CHI.

Local general surgeons were involved in the clinical decision-making, encouraged to take part in all the procedures and supervised in achieving competence with basic paediatric surgical techniques. Attention was paid to evidence-based guidelines in making the most appropriate imaging for a specific paediatric condition and to the management of complex conditions before referral to a specialist centre.

### Ethical consideration

All the procedures were performed under the 1964 Helsinki declaration and its later amendments or comparable ethical standards.

## Results

Cases handled by the visiting team are listed in Table 1. Common conditions of non-specialist level were usually not submitted to the visiting team unless presented unusual clinical features. They are not included in the list.

**TABLE 1:** Paediatric surgical cases observed in 27 weeks at the Consolata Hospital Ikonda.

Diagnosis	Number	Type	Previous procedures and outcome
**General paediatric surgery 360 diagnoses in 316 patients**			
Hernias	210	111 Umbil. (2 perforated) 84 Inguinal (2 perforated) 15 Congenital hydrocele	13 (recurrences)
Undescended testis (mean age 6.2 years, range 2 months–17 years, SD 4.81)	61	-	6 (recurrences)
Phimosis	9	-	2 (recurrences)
Skin lesions: Angiomas, branchial remnants, cysts	63	-	5 (recurrences)
*Abdominal diseases*			
Intussusception	1	-	-
Perforated appendix	2	-	-
Rectal prolapse	4	-	-
Ingested foreign body	1	-	-
Gallstones	3	-	-
*Urology*			
Bladder stones	1	-	-
Urachal cysts	1	-	-
**Specialist paediatric surgery 90 diagnoses in 90 patients**			
*Urology*			
Urethral valves	3	-	-
Vesico ureteral reflux	1	-	-
Bladder diverticulum	1	-	-
Neurogenic bladder	4	-	-
Ambiguous genitalia	4	-	-
Solid neoplasm (all in advanced stage of disease)	10	Wilms tumour 5	-
		Abdominal lymphoma 2	-
		Hepatoblastoma 2	-
		Neuroblastoma 1	-
Anorectal malformations (ARM)	11	H type recto-vaginal fistula 1	-
		Rectovestibular fistula 5	-
		Anteriorised anus 2	-
		Recto bulbar fistula 3	4 (3 Loop colostomy/1 failed PSARP)
Hischsprung’s disease (HSCR)	8	-	4 Loop colostomy
Oesophageal achalasia	1	-	-
Choledochal cyst	1	-	-
Exstrophyc bladder	1	-	-
Hypospadias (mean age 6 years, range 2 months–17 years, SD 57.4)	45	Midshaft or proximal 13 distal 32	5 (all post-operative fistulas)

SD, standard deviation; PSARP, Posterior Sagittal Ano Recto Plasty.

Note: 450 pathologies in 406 children, surgery planned in 378 (14 re do) 355 procedures done on 337 patients, including 16 emergencies.

Among general paediatric surgery cases, simple inguinal herniorrhaphy, instead of herniotomy, was done to prevent recurrences and complications. An earlier approach to undescended testis to avoid testicular damage was advised. Among specialist pediatric surgical cases, hypospadias represented the most common urological disease, and fistulas from previous surgery were not uncommon. The visiting team introduced the local staff to a few simple techniques to manage frequent distal types but discouraged surgery at district level for midshaft and proximal hypospadias.

Loop colostomies for anorectal malformations (ARM) and Hischsprung’s disease (HSCR) were routinely fashioned at the CHI before surgical outreach. Distal colonic faecal impaction or prolapsed loop regularly occurred. A divided stoma was recommended for all new cases, and a conversion from loop colostomy was done before definitive treatment in three ARM and four HSCR cases. Females who could easily pass stools at birth through a rectovestibular fistula were left without a colostomy until later definitive ARM repair to reduce stoma-related family discomfort and prevent distal bowel hypoplasia because of a long-term exclusion. Any blind perineal exploration of ARM cases at birth was discouraged.

Some cases are not listed in Table 1 (two suspected HSCR, one ARM, two posterior urethral valves (PUV), two omphalocele, one atresia, one splenic cyst) and were managed autonomously by local surgeons, during 2019, under the assistance of distant consultation with the visiting team. One of the omphalocele cases and the intestinal atresia case, which could not be transferred to a tertiary centre, died after surgery because of a poor post-operative management.

## Discussion

There is an intense debate on solutions to face the shortage of paediatric surgeons in LICs.^7,8,9,10^ Revisiting surgical residents’ academic curriculum could be the first step.^11^ General surgeons going to practise on paediatric cases in a small centre need only a basic paediatric training (Level-One – General Paediatric Surgery). They should deal with elective conditions such as herniotomy, circumcision, minor soft tissue and orchidopexy for palpable undescended testis or urgent cases such as appendectomy, colostomy, acute scrotum, abscesses and lifesaving trauma surgery. Close connections with specialist tertiary centres warrant safe referral of specialist cases according to shared guidelines and protocols.

International humanitarian outreach by short-term self-contained missions is usually more focused on relieving the burden of diseases than on teaching.^12^ Only in recent years, more attention has been devoted to supporting the training of surgeons and other health workers on paediatric case management.^5^ In Tanzania, a ‘south-to-south’ initiative from local academic institutions as in other LICs^6^ to extend the paediatric surgical coverage to district health facilities is still hard to support by the small local specialists’ workforce. Our outreach aimed to contribute to improving the level of care in a district hospital located more than 800 km away from the nearest specialist centre.

Results of our visits to CHI were encouraging. Although CHI admission records did not document in the past a high paediatric surgical caseload, a hidden demand was unveiled in the Makete District. This confirmed other experiences^13^ about the need for developing paediatric surgical capacities. Awareness of paediatric-specific surgical needs^14^ appeared to increase amongst local providers following surgical outreach. Introducing an earlier approach by a simple herniotomy technique to inguinal hernia,^15^ reducing the mean age at surgery of undescended testis^16^ and adequate approach to hypospadias^17^ may be considered to be significant steps in improving the standard of paediatric surgical care. Current practices of colostomy fashioning (like loop colostomy) were also revised, and a divided stoma following updated technical guidelines^18^ was reccomanded. Although obstructions from congenital colorectal diseases (ARM and HSCR) are a very common neonatal condition in Africa^19,20^ and management in a district hospital is limited to an emergency diversion, colostomy fashioning by non-paediatric surgeons is reported to be associated with a high rate of complications^21^ making definitive treatment more complex.

## Lessons learnt

Surgical outreach and capacity building at the district hospital level could be a possible answer to the unmet paediatric surgical needs of children living in rural areas. A participatory approach, friendly environment, mutual trust and a shared view of quality-based healthcare inspired humanitarian outreach at the CHI. Nevertheless, interest and motivation of general surgeons to extend their paediatric knowledge and to increase self-confidence in their paediatric clinical and surgical skills must be increased. Elective cases are frequently left to foreign specialists, and arranging a waiting list for the visitors continues to be more attractive for local surgeons than starting an autonomous unsupervised activity. Management of complex scenarios by distant consultation was only recently begun at the CHI. This is a supplementary resource to be further implemented. A more substantial commitment by local health providers to increase their paediatric surgical skills and a longer ‘time on target’ of outreach is still needed to achieve long-lasting results.

## References

[1] Bickler SW, Telfer ML, Sanno-Duanda B Need for pediatric surgical care in an urban area of The Gambia. Trop Doct. 2003 4;33(2):91–4. 10.1177/004947550303300212 12680542

[2] The Royal College of Surgeons of England Standards for children’s surgery. London: Publisher RCSENG – Professional Standards and Regulation; 2013.

[3] Krishnaswami S, Nwomeh BC, Ameh EA The pediatric surgery workforce in low- and middle-income countries: Problems and priorities. Semin Pediatr Surg. 2016 2;25(1):32–42. 10.1053/j.sempedsurg.2015.09.007 26831136

[4] Philipo GS, Nagraj S, Bokhary ZM, Lakhoo K Lessons from developing, implementing and sustaining a participatory partnership for children’s surgical care in Tanzania. BMJ Glob Health. 2020 3 17;5(3):e002118 10.1136/bmjgh-2019-002118 PMC7078648 32206345

[5] Mitchell KB, Giiti G, Kotecha V, Chandika A, Pryor KO, Härtl R Gilyoma surgical education at Weill Bugando Medical Centre: Supplementing surgical training and investing in local health care providers. J Can J Surg. 2013 6;56(3):199–203. 10.1503/cjs.028911 PMC3672435 23484467

[6] Madhuri V, Stewart RJ, Lakhoo K Training of children’s surgical teams at district level in low- and middle-income countries (LMIC): From concept to reality; A south to south initiative. Int J Surg Glob Health. 2019;2:e08 10.1097/GH9.0000000000000008

[7] World Health Organization Surgical care systems strengthening: Developing national surgical, obstetric, and anaesthesia plans. Geneve: WHO, and Boston: Harward Medical School; 2017.

[8] Poenaru D The burden of pediatric surgical disease in low-resource settings: Discovering it, measuring it, and addressing it. J Pediatr Surg. 2016;51(2):216–222. 10.1016/j.jpedsurg.2015.10.065 26655214

[9] Derbew M Pediatric surgery in Eastern Africa: The unmet need. J Pediatr Surg. 2019 1;54(1):21–26. 10.1016/j.jpedsurg.2018.10.028 30454942

[10] Royal College of Surgeons of England, the Royal College of Surgeons of Edinburgh, the Royal College of Physicians and Surgeons of Glasgow, and the Surgical Forum, Working together to improve the local delivery of the General Surgery of Childhood – Statement of intent, London: FSSA Publications & Surveys; 2018.

[11] Raveenthiran V, Sarin YK Pediatric surgical training in India: Proposal of a new scheme. J Indian Assoc Pediatr Surg. 2006;11(2):203–207. 10.4103/0971-9261.25936

[12] Kynes JM, Zeigler L, McQueen K Surgical outreach for children by International Humanitarian Organizations: A review. Children (Basel). 2017 6 28;4(7):53 10.3390/children4070053 PMC5532545 28657589

[13] Butler EK, Tran TM, Nagarajan N, et al Epidemiology of pediatric surgical needs in low-income countries. PLoS One. 2017 3 3;12(3):e0170968 10.1371/journal.pone.0170968 28257418 PMC5336197

[14] Tam PKH, Davenport M, Chan IHY, Numanoglu A, Hoebeke P, Diamond DA Long-term implications and global impact of Paediatric surgery. Lancet. 2017 9 9;390(10099):1012–1014. 10.1016/S0140-6736(17)32341-3 28901925

[15] Ohene-Yeboah M, Abantanga FA Inguinal hernia disease in Africa: A common but neglected surgical condition. West Afr J Med. 2011;30(2):77–83. 21984452

[16] David OO, Iyekoretin E Undescended testes in a developing country: A study of the management of 71 patients. Afr J Paediatr Surg. 2008 Jan-Jun;5(1):11–14. 10.4103/0189-6725.41629 19858656

[17] Ogundoyin OO, Olulana DI, Lawal TA, Ademola SA Management of hypospadias in a resource-poor setting: The Ibadan experience. Nigerian J Plast Surg. 2017;13(2):40–44. 10.4103/njps.njps_5_17

[18] Pena A, Migotto-Krieger M, Levitt MA Colostomy in anorectal malformations: A procedure with serious but preventable complications. J Pediatr Surg. 2006;41(4):748–756. 10.1016/j.jpedsurg.2005.12.021 16567188

[19] Poenaru D, Borgstein E, Numanoglu A, Azzie G Caring for children with colorectal disease in the context of limited resources. Semin Pediatr Surg. 2010 5;19(2):118–127. 10.1053/j.sempedsurg.2009.11.017 20307848

[20] Moore S, Alexander A, Sidler D, et al The spectrum of anorectal malformations in Africa. Pediatr Surg Int. 2008;24:677–683. 10.1007/s00383-008-2131-y 18386020

[21] Lawal TA Overview of anorectal malformations in Africa. Front Surg. 2019 3 5;6:7 10.3389/fsurg.2019.00007 30891450 PMC6411760

